# Development of a New Tablet-Based Cognitive Screening Test for Community-Dwelling Older Adults

**DOI:** 10.7759/cureus.111025

**Published:** 2026-06-17

**Authors:** Masayuki Satoh, Shin-ichiro Maeshima, Nao Hatakeyama, Yoshinori Ota

**Affiliations:** 1 Neurology, Center for Comprehensive Care and Research on Memory Disorders, National Center for Geriatrics and Gerontology, Ōbu, JPN; 2 Physical Medicine and Rehabilitation, Education and Innovation Center for Geriatrics and Gerontology, National Center for Geriatrics and Gerontology, Ōbu, JPN; 3 Internal Medicine, Center for Comprehensive Care and Research on Memory Disorders, National Center for Geriatrics and Gerontology, Ōbu, JPN; 4 Research and Development, Research Institute of Brain Activation, Tokyo, JPN

**Keywords:** cognitive assessment of community-dwelling older adults, cognitive decline, japanese geriatrics, japanese version of mini-mental state examination, tablet

## Abstract

Background: Early detection of cognitive decline in community-dwelling older adults requires accessible, automated screening tools that minimize the burden on healthcare personnel. To address this, we developed a novel, tablet-based cognitive screening tool that might be administered within the Specific Health Checkups (SHC) framework and used by *Minsei-iin *(Commissioned Volunteer Community Welfare Officers)*.*

Methods: We developed "Cognitive Assessment for Community-Dwelling Older Adults" (CANDIDE), a tablet-based cognitive test designed to evaluate multiple cognitive domains within eight minutes. Over a period of six months, outpatients at our hospital's memory clinic (Center for Comprehensive Care and Research on Memory Disorders, National Center for Geriatrics and Gerontology, Ōbu, Japan) underwent both the CANDIDE and the Japanese version of the Mini-Mental State Examination (MMSE-J). To accommodate diverse sensory needs, instructions were presented via both auditory and visual modalities. The statistical correlation between the two assessments was evaluated to determine the provisional cutoff score of the new tool.

Results: A total of 70 participants were enrolled (34 males, 36 females; mean age: 80.9 ± 6.5 years; mean education years: 11.6 ± 2.2 years). A strong positive correlation was observed between the total scores of the CANDIDE and the MMSE-J (r = 0.721, p < 0.001). Based on linear regression, the standard MMSE-J cutoff score of 24 corresponded to a CANDIDE score of 21.2.

Conclusion: The CANDIDE might be a feasible tool for screening of cognitive decline in individuals aged 40-74. Further investigation is warranted to validate its clinical utility and cost-effectiveness within the annual SHC framework, as well as its efficacy as an objective assessment tool for *Minsei-iin*.

## Introduction

It is increasingly recognized that dementia is, to a certain extent, preventable. A landmark report indicated that modifying 14 lifestyle risk factors could potentially prevent or delay nearly 40% of dementia cases [1]. In a Japanese longitudinal study of community-dwelling older adults with mild cognitive impairment (MCI), approximately 30% and 18% of participants reverted to normal cognition within 5 and 10 years, respectively [2]. These findings suggest that the effective management of lifestyle-related diseases and the promotion of health-seeking behaviors are critical in facilitating cognitive recovery. Furthermore, following the recent clinical introduction of anti-amyloid-beta antibodies for Alzheimer’s disease (AD) in Japan, the clinical imperative for the early detection of cognitive decline has never been greater [3].

Under Japan's national health policy, Specific Health Checkups (SHC) are conducted annually for individuals aged 40 to 74, as mandated by the Act on Assurance of Medical Care for Elderly People (Act No. 80 of 1982, revised in 2008). While these screenings effectively target metabolic syndrome and cardiovascular risk factors, such as type 2 diabetes, hypertension, and dyslipidemia, cognitive screening is not currently a mandatory component. Consequently, cognitive assessments are omitted by most municipalities. According to the Ministry of Health, Labour and Welfare, only 10%-15% of municipalities conducted cognitive assessments in 2020 [4]. Even in Tokyo, recognized for its advanced local governance, only 35% of municipalities had implemented independent dementia screening programs by 2022 [5]. The primary barriers to widespread implementation include a lack of brief, validated screening tools and a shortage of trained personnel to administer traditional neuropsychological tests [6].

In the Japanese community-based integrated care system, *Minsei-iin* (Commissioned Volunteer Community Welfare Officers), legally defined under the *Minsei-iin* Law (Law on Commissioned Welfare Volunteers, Act No. 198 of 1948), play a pivotal role. As part-time, unpaid civil servants, they monitor the well-being of vulnerable elderly residents and serve as a vital liaison to medical and social services. Although some municipalities have introduced cognitive checklists for these volunteers (e.g., Health and Welfare Bureau, Kanazawa) [7], such tools often rely on subjective impressions and lack clinical rigor. The development of an accessible, affordable, and objective cognitive test would bridge this gap, ensuring early detection and timely intervention.

We previously developed an online cognitive assessment (Brain Assessment, or BA), which demonstrated moderate correlations with established in-person neuropsychological tests [8-10]. However, the BA was primarily designed for the working population (aged 40+) to track longitudinal changes relative to normal aging; its difficulty level makes it less suitable for screening community-dwelling older adults. While various digital cognitive tools have gained popularity in Japan, they are not universally suitable for screening community-dwelling older populations. To address this issue, we developed a novel, tablet-based cognitive screening tool, which might be administered for use in the SHC framework and by *Minsei-iin*. With the ultimate goal of deploying this system in community health checkups, the primary and secondary objectives of the present study were to evaluate this brief tool among outpatients of our memory clinic and suggest its provisional cutoff score, respectively, by examining its correlation with the standard in-person screening test in a clinical setting.

## Materials and methods

Development of the new cognitive test

The newly developed tool, "Cognitive Assessment for Community-Dwelling Older Adults" (CANDIDE), was designed based on the following criteria: (i) tablet-based administration; (ii) a completion time of seven to eight minutes; (iii) touch-pen interface; (iv) multi-domain assessment (memory, orientation, calculation, visuospatial, and executive function); (v) dual-modality instructions (auditory and visual) to accommodate hearing-impaired subjects; and (vi) instantaneous result processing and cloud storage. One point was allocated for each correct response, with the total score representing the sum of points from all questions. The hardware and software specifications of the tablet-based cognitive screening system used in this study are summarized in Table 1.

**Table 1:** Specifications IPS, in-plane switching; MDM, mobile device management; RAM, random access memory

Category	Specification	Manufacture/developer, location
Hardware specifications		
Device model	Lenovo Tab K11 (model: ZADG0015JP)	Lenovo Japan LLC, Tokyo
Display	10.95-inch IPS display, 1920 x 1200 pixels	
Processor	MediaTek Helio G88	MediaTek Inc., Hsinchu, Taiwan
Memory	4 GB RAM	Lenovo Japan LLC, Tokyo
Storage	64 GB	
Input method	Capacitive touchscreen	
Operation system	Android 14	Google, Mountain View, California, USA
Web application		
Access control application	Web-based cognitive assessment system by RIBA	Research Institute of Brain Activation Co., Ltd. (RIBA), Tokyo
	Proprietary MDM application used to provide controlled access to the assessment system	

Study participants

The study participants were consecutive outpatients who visited our memory clinic (Center for Comprehensive Care and Research on Memory Disorders, National Center for Geriatrics and Gerontology, Ōbu, Japan) and were treated (by MS, SM, NH) between September 2025 and March 2026. The inclusion criteria were as follows: (a) first-time outpatients visiting our memory clinic, (b) presentation of stable physical and psychological conditions, (c) adequate hearing and visual acuity required to complete the tasks, and (d) provision of written informed consent to participate in this study. Conversely, participants were excluded if they met any of the following criteria: (a) unstable physical or psychological conditions, (b) impaired consciousness, or (c) inability to complete the assessment.

Assessments

Participants underwent both the CANDIDE and the Japanese version of the Mini-Mental State Examination (MMSE-J) [11]. To minimize bias, the tests were administered one month apart, with the order of administration randomized for each patient. The evaluators, namely, authors MS, SM, and NH, knew the prior test results. Participants were diagnosed with MCI in accordance with the Petersen criteria [12].

Statistical analyses

Data normality was confirmed, and Pearson’s correlation coefficient (r) was used to evaluate the relationship between scores. Correlation strengths were defined as small (0.0-0.2), fair (0.2-0.4), moderate (0.4-0.7), and strong (0.7-1.0). Mean values were compared using Welch’s t-test, with significance set at p < 0.05. All analyses were performed using IBM SPSS Statistics, version 29 (IBM Corp., Armonk, NY).

Ethical consideration

This study received approval from the National Center for Geriatrics and Gerontology Research Ethics Committee (approval no. 1814) and was conducted in accordance with the Declaration of Helsinki (1975). Following the explanation about this study by the author, each participant gave written consent by writing their name and address on the consent form.

## Results

**Figure 1:**
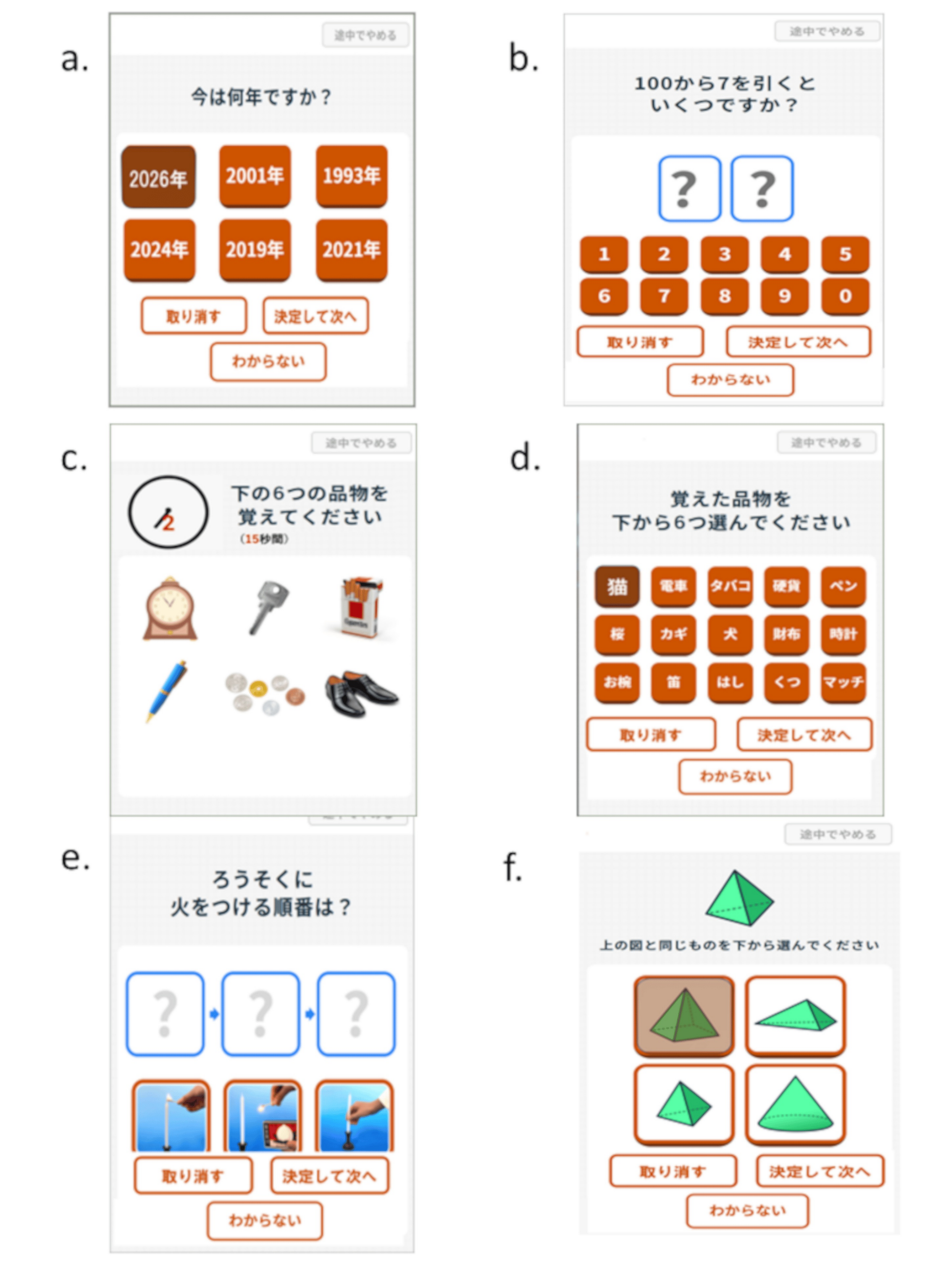
Examples of tablet screens a. “What year is it now?”. b. “What is 100 minus 7?”. c. “Memorize the following six objects”. d. “Select six objects you have memorized in the preceding screen”. e. “Arrange the order of lighting the candle”. f. “Select the same figure as that represented above”. The screen c is represented for 15 seconds, and then automatically moved to the next screen d. Three buttons located at the bottom of the screen are “Cancel”, “Decide and go to next screen”, and “Cannot answer”. The right upper box means “Discontinue the test.”

The components of the CANDIDE are summarized in Table 2. The test assessed both verbal and visual memory, with a maximum total score of 30, aligning it with the MMSE [13] and the Revised Hasegawa Dementia Scale (HDS-R) [14]. Representative tablet interface screens are shown in Figure 1. Participants were instructed to respond to each item by touching the screen with a stylus, and they were able to perform the test without prior training. If participants did not know the answer, they could select the “Cannot answer” button on the screen to proceed to the next item. If participants replied with a wrong answer, they could cancel it by touching the “Cancel” button on the screen. The operation of the tablet was easy because participants were only required to touch the screen. They could complete the CANDIDE without or with only a little help from the examiner, so tablet-use difficulty with respect to each participant did not influence the results.

**Table 2:** Contents and examples of questions of the Cognitive Assessment for Community-Dwelling Older Adults (CANDIDE)

Contents	Examples	Number of questions	Score
Time orientation	What day is it today?	5	5
Immediate recall	Cherry, cat, train	3	3
Calculation	100 - 7 = ?	5	5
Reverse repeat	Reverse the order of the following numbers: 6 - 8 - 2	2	2
Delayed recall	Recall the 3 words which you had remembered.	3	3
Naming	What is this? (pencil, watch)	2	2
Immediate recall of six objects	Select six objects which you have seen immediately before.	6	6
Praxis	Arrange three photos by the order to do.	2	2
Visuospatial function	Select the same figure.	2	2
Total			30

**Figure 2:**
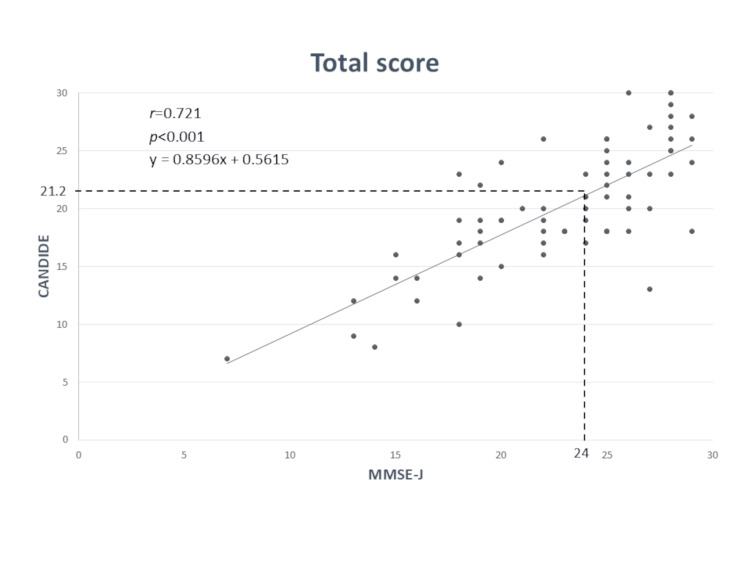
Correlation between total scores of the MMSE-J and CANDIDE A cutoff of 24 in the MMSE-J corresponds to 21.2 in the CANDIDE. CANDIDE, Cognitive Assessment for Community-Dwelling Older Adults; MMSE-J, Japanese version of the Mini-Mental State Examination

Between September 2025 and March 2026, 70 outpatients were enrolled (34 male and 36 female patients; mean age, 80.9 ± 6.5 years; mean education years: 11.6 ± 2.2 years). Patient characteristics are detailed in Table 3. A strong positive correlation was observed between the total scores of the CANDIDE and the MMSE-J (r = 0.721, p < 0.001; Figure 2).

**Table 3:** Participants' characteristics F, female; M, male; MMSE-J, Japanese version of the Mini-Mental State Examination

	N = 70	M:F = 34:36
Age (years)	80.9 ± 6.5	M: 80.5 ± 6.3
		F: 81.4 ± 6.8
Education (years)	11.6 ± 2.2	M: 12.0 ± 2.4
		F: 11.1 ± 2.0
MMSE-J score	22.8 ± 4.7	M: 22.6 ± 4.7
		F: 23.0 ± 4.8

Comparative mean scores for sub-items are presented in Table 4. Notably, while MMSE-J scores were significantly higher for calculation, CANDIDE scores were significantly higher for delayed recall. The mean administration time for the CANDIDE was 414 ± 156 seconds (approximately seven minutes). Based on the formula of linear regression, an MMSE-J cutoff of 24/25 corresponds to a CANDIDE score of 21.2 (Figure 2). Thus, a CANDIDE score of ≤21 might suggest cognitive decline. For the 25 participants diagnosed with MCI, the mean scores were 21.4 ± 3.9 for the CANDIDE and 24.4 ± 3.1 for the MMSE-J.

**Table 4:** Results of tests CANDIDE, Cognitive Assessment for Community-Dwelling Older Adults; MMSE-J, Japanese version of the Mini-Mental State Examination

	MMSE-J	CANDIDE	p-value
Total score	22.8 ± 4.7	20.2 ± 5.3	0.002
Date	3.6 ± 1.5	3.7 ± 1.4	0.47
Immediate recall	2.9 ± 0.50	2.9 ± 0.30	0.65
Calculation	3.4 ± 1.4	2.5 ± 1.7	0.001
Delayed recall	1.0 ± 1.1	2.3 ± 0.90	<0.001

## Discussion

The CANDIDE demonstrated a strong correlation with the MMSE-J, and its performance time was within the target range for high-volume clinical or community screenings. Furthermore, the dual-modality instruction system ensured the test was accessible to participants with hearing impairment. The objectives of this study were to develop a novel screening tool and suggest its provisional cutoff score. Although this study is limited by a small sample size, a single-center, memory-clinic cohort, and the absence of cognitively unimpaired community controls, future large-scale implementation in community health checkups will allow for a more rigorous evaluation of diagnostic parameters. Furthermore, the capacity of this tool to differentiate between specific etiologies, such as AD, vascular dementia, and dementia with Lewy bodies, warrants further investigation. Ultimately, the present study might suggest the utility of the CANDIDE in clinical and community settings.

The observed discrepancies in sub-item scores (calculation and delayed recall) likely stem from differences in task modality. In the MMSE-J calculation task (serial sevens), subjects respond orally. In the CANDIDE, subjects must hold the calculated value in working memory while navigating the tablet interface to input the answer; this increased cognitive load may explain the lower calculation scores. Conversely, for delayed recall, the MMSE-J utilizes free recall, whereas the CANDIDE utilizes a recognition task (selecting from nine options), which inherently facilitates better performance.

Table 5 summarizes the primary digital cognitive assessments currently available in Japan [8-10,15,16]. All listed instruments have been reported in international peer-reviewed journals and demonstrate significant correlations with the MMSE. Each test possesses distinct methodological characteristics, including its format, primary target population, administration time, assessed cognitive domains, and reference data.

**Table 5:** Characteristics of computer- and tablet-based cognitive assessments BA, Brain Assessment; CANDIDE, Cognitive Assessment for Community-Dwelling Older Adults; CBB, CogState Brief Battery; MCI, mild cognitive impairment; min., minutes; SHC, Specific Health Checkups; y.o., years-old "〇" represents "included in the test".

Features	CBB	CogEvo	BA	CANDIDE
Test format	Card selection	Mini-games	Cognitive tests	Cognitive tests
Classification	Medical instruments	General use	General use	General use
Target population	Patients with dementia	Community-dwelling elderly people, patients with MCI	Working-age population	Community-dwelling elderly people
Input method	Key tapping	Touch panel	Touch panel	Touch panel
Stimulus modality	Visual	Visual + auditory	Visual + auditory	Visual + auditory
Administration time (min.)	10-20	5-10	Original: 25-30, Short version: 8-10	7-8
Assessed cognitive domains				
Orientation		〇	〇	〇
Verbal memory				
Immediate recall		〇	〇	〇
Delayed recall		〇	〇	〇
Visual memory	〇		〇	〇
Calculation			〇	〇
Naming			〇	〇
Praxis			〇	〇
Visuospatial function		〇	〇	〇
Attention	〇	〇	〇	〇
Executive function (judgment/planning)		〇	〇	〇
Reaction time	〇			
Result presentation	Score, time	Radar chart	Standardized scores (T-scores/Z-scores)	Score
Reference data	118 normal subjects (>55 y.o.) [15]	272 community-dwelling subjects (MCI, dementia patients included), F:M = 246:26 [16]	>5,000 normal subjects (40-85 y.o.), F:M = 2,500:2,500 [8]	70 outpatients of the memory clinic, F:M = 36:34
Primary applications	Clinical trials	Screening + rehabilitation	Annual health checkups by enterprises	SHC, by *Minsei-iin*

Specifically, the CogState Brief Battery (CBB) has an extensive track record in clinical trials, although it requires a relatively long administration time. The CogEvo, while incorporating gamified elements and offering pathways to cognitive training or rehabilitation, is supported by normative data with a female-predominant distribution. In contrast, the Brain Assessment provides comprehensive coverage of cognitive domains, supported by a large-scale, gender-balanced dataset, making it suitable for annual health screenings in the working-age population. Similarly, the CANDIDE covers a broad range of cognitive domains and is designed for screening cognitive decline in older adults.

Selecting the most appropriate instrument requires careful consideration of the clinical objective, the target demographic, and the available time for assessment. In this context, the CANDIDE might hold potential to contribute to health screening and community-based care activities.

We may say that the use of the internet does not represent a barrier for today’s older adults to perform the CANDIDE. According to the Ageing Society 2021 report published by the Cabinet Office in Japan [17], about 74% and 58% of septuagenarians and octogenarians, respectively, utilize the internet, and the rates have increased almost two-three folds compared with the results from 2010 (septuagenarians 39.2%, octogenarians 20.3%) [18]. The utilization of the internet by older adults is increasing yearly, and we expect that more older adults will be able to perform digital cognitive tests more easily in the future.

This study has several limitations. First, the sample size was small, and the cohort consisted of memory clinic outpatients, resulting in a lack of cognitively normal controls. Future community-based implementation will be necessary to validate these findings in the general population. Second, while the CANDIDE was compared with the MMSE-J, ongoing research is evaluating its correlation with the Frontal Assessment Battery (FAB) [19] and logical memory tasks. Third, the sensitivity and specificity for MCI detection will be refined through broader social implementation. Finally, the ultimate goal of this study was to develop a novel screening tool. By multicenter deployment of the CANDIDE within the SHC framework, future studies will be able to establish more precise diagnostic parameters, including sensitivity, specificity, receiver operating characteristic (ROC) curves, area under the curve (AUC), positive and negative predictive values, and optimal cutoff scores, to accurately distinguish cognitively impaired individuals from cognitively unimpaired counterparts. These parameters will potentially demonstrate the utility of this tool in community screening settings. Despite these limitations, the CANDIDE might be a tool for early cognitive screening of older adults, potentially facilitating earlier intervention for dementia.

## Conclusions

We developed a tablet-based cognitive assessment tool (CANDIDE) for older adults. Our results revealed a strong correlation with the MMSE-J, demonstrating the feasibility of this test for administration. We anticipate that the CANDIDE might be useful in health screening and community care activities. Future large-scale studies are required to validate the findings in the general population and establish more precise diagnostic parameters.
